# Intent-Conditioned Diffusion Trajectory Prediction for Proactive Lane-Change Risk Assessment

**DOI:** 10.3390/e28090957

**Published:** 2026-08-26

**Authors:** Lijing Ma, Shaofei Zhang, Wei Zhang, Jiacheng Yin, Yilong Wu

**Affiliations:** 1 School of Automobile and Transportation, Xihua University, Chengdu 610039, China; malijing@xhu.edu.cn (L.M.); zhangshaofei@stu.xhu.edu.cn (S.Z.); yinjiacheng@xhu.edu.cn (J.Y.); wyl18222227054@163.com (Y.W.); 2 College of Intelligent Construction and Manufacturing, Sichuan Technology and Business University, Chengdu 611745, China

**Keywords:** lane-change risk assessment, proactive safety, intent-conditioned diffusion, trajectory prediction, surrogate safety measure

## Abstract

Proactive lane-change risk assessment requires estimating whether an intended maneuver will lead to unsafe interactions before the maneuver is completed. This is challenging in naturalistic driving data because actual crashes are extremely rare, and binary collision labels provide little discriminative information for learning risk. We propose IntentDiff, an intent-conditioned diffusion framework for proactive lane-change risk assessment. The framework uses predicted future trajectories as the basis for risk estimation. A vectorized scene context learning module combines a VectorNet backbone with a Vector Quantized Variational Autoencoder (VQ-VAE) to map agent–map interactions into discrete intent codes. These codes organize complex traffic situations into interpretable intent prototypes and provide semantic guidance for trajectory generation. Conditioned on the learned intent code, a diffusion model generates kinematically consistent multimodal trajectories of the target vehicle. On the forecast trajectories, Monte Carlo rear-end risk is evaluated against the four bounding vehicles and fused into a Lane-Change Risk Index (LCRI). On the highD dataset, the framework attains an average displacement error of 0.42 m over a 5-s horizon. The forecast-based LCRI agrees closely with the index computed from realized future trajectories, indicating that most high-risk lane changes can be identified before the maneuver is completed. Grouping LCRI by intent code further reveals systematic variation in risk across lane-change maneuvers, suggesting that the learned codebook captures risk-relevant interaction patterns in addition to maneuver semantics.

## 1. Introduction

Driving risk assessment is an essential function of intelligent vehicles, as safety-critical interactions must be recognized before they develop into crashes. This requirement is particularly important in highway lane-changing scenarios. A small longitudinal or lateral mismatch between a lane-changing vehicle and its surrounding vehicles can rapidly lead to a rear-end or sideswipe conflict. For advanced driver assistance and autonomous decision-making, the central question is therefore not whether a completed maneuver was risky, but whether the future risk of an intended lane change can be estimated early enough to support preventive action. Recent studies [1,2] emphasize that this problem is difficult because risk is not determined solely by the target vehicle but arises from the coupled motion of the target vehicle, neighboring vehicles, road geometry, and the future responses of all interacting agents.

Research on driving risk assessment has gradually moved from instantaneous state judgment to future-oriented risk inference. Since actual crashes are extremely rare in naturalistic traffic, classical surrogate safety measures (SSM) replace sparse and delayed crash records with measurable conflict indicators derived from vehicle proximity, time margins, required braking, or hypothetical collision severity [3,4]. These indicators are commonly grouped into time-based measures such as Time to Collision (TTC), deceleration- and distance-based measures such as the Deceleration Rate to Avoid a Crash (DRAC), and severity-oriented measures [5]. Earlier probabilistic scene-analysis and driver-behavior models have likewise linked predicted motion to collision probability and decision-making risk [6,7]. Although interpretable and computationally efficient, these measures often rely on fixed thresholds, pairwise interaction assumptions, and scenario-specific definitions, which limits their use when several vehicles interact and when both longitudinal and lateral conflicts must be considered.

Risk assessment is particularly challenging in multi-vehicle highway interactions, where the risk state depends jointly on the target vehicle and several surrounding vehicles. Xiong et al. [2] divide multi-vehicle risk assessment methods into state-inference-based and trajectory-prediction-based approaches. Most existing lane-change risk studies fall on the state-inference side as they map historical features directly to a risk state or compute surrogate measures on observed trajectories, without retaining the predicted future segment [8,9,10]. Representative examples include the rear-end crash risk index of Shangguan et al. [8], which combines Monte Carlo crash occurrence with collision severity and was later aggregated across the four key surrounding vehicles into a Lane-Change Risk Index (LCRI) through a fault-tree formulation [11], as well as recent generalized surrogate safety models learned from large-scale naturalistic data without crash labels [12]. Trajectory-prediction-based methods instead forecast the future motion distribution and then evaluate collision probability or severity over the prediction horizon. Although more costly, they account for the future spatial relations among interacting vehicles and can detect collision risk earlier and more accurately [2]. Recent studies illustrate this trend by coupling multi-vehicle trajectory prediction with collision probability, collision intensity, or aggregated lane-change risk indices [13,14,15], and risk-aware predictors have begun to incorporate collision risk into the prediction objective itself [16]. This direction naturally connects risk assessment with trajectory prediction, because forecast trajectories make the source and timing of a potential conflict observable before the maneuver unfolds. However, it requires a forecasting framework whose output is both suitable for proactive risk computation and semantically organized enough to explain why some lane-change situations are riskier than others.

Driving intention recognition provides the semantic ingredient for such forecasts. An intention denotes a deliberate, goal-level decision formed earlier in the timeline than the behavior that executes it, so recognizing it offers an earlier cue for safe decision-making than forecasting the trajectory alone [17,18]. Methodologically, intention recognition has evolved from inferring discrete maneuvers from vehicle kinematics with classical classifiers toward learning spatial and temporal features directly from data. This includes combined CNN–LSTM architectures for lane-change anticipation [19], convolutional social pooling [20] and attention-based models [21] for capturing the interaction context, graph-based models that dynamically update relational edges [22], and transformer-based predictors that jointly model maneuver and trajectory in a multimodal form [23,24]. More recent models increasingly couple intention with trajectory generation, for example by integrating intention awareness into diffusion-based prediction [25] or by obtaining intention-aware motion modes through contrastive clustering [26]. These studies indicate that intention information improves motion prediction. In most designs, however, intention enters as a supervised label, an auxiliary branch, or an input token. Such usage helps the predictor select a plausible mode, yet it does not yield an interpretable representation that can be tied to downstream risk.

The forecasting task itself is inherently generative, because a single observed history may correspond to multiple plausible futures. Lane-change futures are multimodal, interaction-dependent, and constrained by road topology and vehicle kinematics [18,27,28,29,30], which makes deterministic point prediction insufficient. Early generative approaches used conditional variational autoencoders that sample latent variables to generate alternative future hypotheses [31] and adversarial models that encourage socially compliant outputs [32]. Conditional VAEs, however, are prone to posterior collapse and tend to regress toward an average path. GANs are hard to train and frequently exhibit mode collapse. The latent representations of both families are continuous and entangled, which makes it difficult to identify which dimensions correspond to a particular driving intent. Diffusion models have recently emerged as a more stable alternative with superior mode coverage, modeling trajectory generation as a gradual denoising process [33], accelerating inference through leapfrog strategies [34], and incorporating feasibility constraints by conditioning on discrete traffic contexts generated by a VQ-VAE [35]. Nevertheless, standard diffusion predictors typically operate in a continuous latent space and lack explicit semantic control. Hence, the generation process is often blind and may require a large number of samples to cover the ground-truth mode. Many also rely on rasterized maps or time sequences that lose fine-grained topological information compared with vectorized representations [36].

In summary, existing studies have established three important foundations for proactive lane-change risk assessment. Intention recognition provides early semantic cues about future behavior, and generative trajectory prediction represents multiple plausible futures under interaction and road constraints. Surrogate safety measures offer interpretable risk indicators when crash labels are unavailable. However, the links among these components remain weak. Intention-aware predictors often lack a risk-oriented interpretation of their latent representations. Generative predictors produce futures without exposing semantically meaningful intent structures. Most risk assessment methods compute risk from observed trajectories or historical features rather than from interpretable predicted futures. The missing link is therefore a predictor that generates feasible futures, exposes the intent structure behind them, and allows the downstream risk index to be traced back to that structure.

To address these limitations, this paper proposes IntentDiff, an intent-conditioned diffusion framework for proactive lane-change risk assessment. The proposed framework uses predicted futures as an explicit basis for evaluating how lane-change risk will evolve before the maneuver unfolds. The forecast is feasible, multimodal, and associated with discrete intent-related prototypes, so that the resulting risk index can be analyzed in terms of the interaction pattern that produces it. From an information-theoretic viewpoint, the framework compresses the high-dimensional traffic scene into a discrete latent bottleneck that retains the maneuver- and risk-relevant information, a property we quantify through the entropy, perplexity, and mutual information of the learned codebook. The main contributions of this paper are summarized as follows.

- A vectorized scene context learning module is developed to provide an interpretable intent basis for risk assessment. The module combines a VectorNet backbone with a Vector Quantized Variational Autoencoder (VQ-VAE), mapping agent–map interactions onto a discrete codebook. These codes represent maneuver-associated intent prototypes, allowing complex lane-change situations to be organized into risk-relevant interaction patterns.
- An intent-conditioned diffusion trajectory generator is proposed to support proactive risk inference. The learned discrete intent code serves as a high-level prior during denoising, while the model generates acceleration and yaw-rate sequences, which are integrated through a kinematic layer. The resulting trajectories are diverse, feasible, and associated with explicit intent prototypes, which makes them suitable for downstream risk computation.
- A forecast-based Lane-Change Risk Index (LCRI) analysis is constructed to evaluate future collision risk prior to the maneuver. For the generated trajectories, Monte Carlo rear-end risk is evaluated against the four bounding vehicles and then fused across the lane-change conflict structure. This allows the framework to quantify proactive risk and to reveal how different intent prototypes correspond to different levels of lane-change risk.

The remainder of this paper is organized as follows. Section 2 details the methodology, including the vectorized scene context learning, the intent-conditioned diffusion model, and the predictive lane-change risk assessment. Section 3 introduces the experimental setup. Section 4 presents the trajectory prediction and the predictive risk assessment results. Finally, Section 5 concludes the paper.

## 2. Methodology

**Figure 1:**
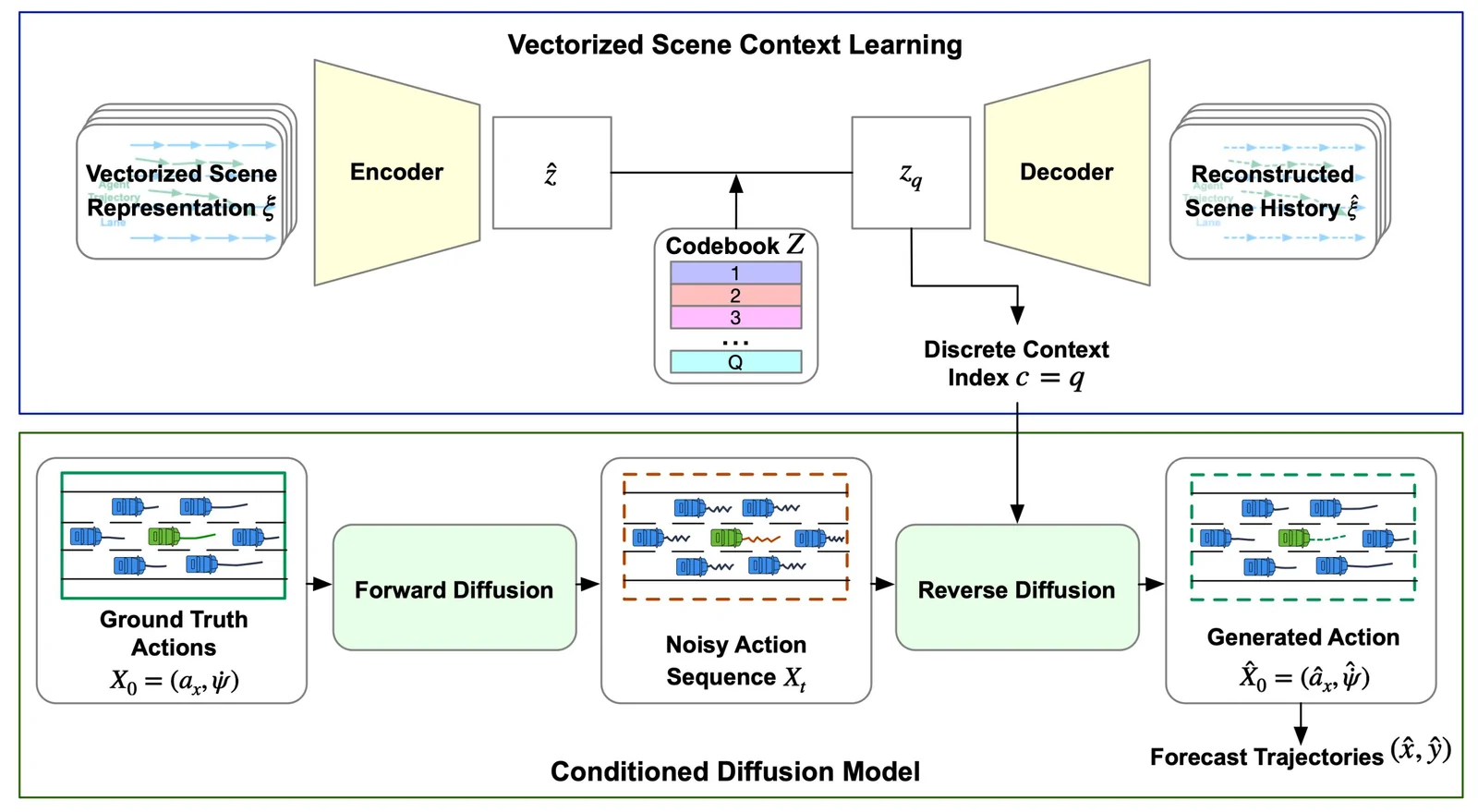
IntentDiff architecture.

This section introduces the trajectory-prediction-based proactive lane-change risk assessment. The framework consists of three functional components: vectorized scene context learning, intent-conditioned diffusion trajectory generation, and predictive lane-change risk assessment. As illustrated in the IntentDiff architecture (Figure 1), a VectorNet-based VQ-VAE encodes heterogeneous agent–map interactions into a discrete latent code associated with maneuver-level driving intents. Subsequently, a 2D U-Net DDPM, conditioned on this discrete intent code, generates dynamically feasible target-vehicle motion control sequences, which are integrated via a kinematic model to produce final forecast trajectories. Finally, the predicted target-vehicle trajectories are evaluated against the four bounding vehicles through a Monte Carlo rear-end risk model and fused into the Lane-Change Risk Index (LCRI). The remaining subsections detail the formal problem statement, scene context learning, conditional trajectory generation, and predictive risk assessment.

### 2.1. Problem Statement

The trajectory prediction task is formulated as the problem of generating the joint probability distribution over the target agent’s future states, conditioned on the historical context of the dynamic traffic scene. Let the dataset $D={\left\{\left(\xi^{\left(m\right)},Y^{\left(m\right)},s^{\left(m\right)}\right)\right\}}_{m=1}^{M}$ be composed of *M* distinct data samples. Each data sample holds the vectorized traffic scenario observations $\xi^{\left(m\right)}$, the future trajectory $Y^{\left(m\right)}$, and a maneuver label $s^{\left(m\right)}$. The scene observation $\xi^{\left(m\right)}$ encompasses the dynamic states of *N* interacting agents (including the ego vehicle and its neighbors) and the static high-definition map vectors over a historical horizon of length $T_{obs}$. Unlike raster-based methods that use fixed-size tensors, $\xi^{\left(m\right)}$ is represented as a set of polylines to preserve geometric sparsity. The target future trajectory is defined as $Y^{\left(m\right)}\in R^{T_{pred}\times 2}$, representing the 2D global coordinates over the prediction horizon $T_{pred}$. To support intent-aware prediction, each sample includes a maneuver class $s^{\left(m\right)}$, which is a one-hot encoded vector representing the driving intention. Specifically, $s^{\left(m\right)}$ indicates whether the future trajectory $Y^{\left(m\right)}$ corresponds to a Lane Change Left (LCL), Lane Change Right (LCR), or Keep Lane (KL) maneuver.

The objective is to predict the future trajectory $\hat{Y}$ for the target agent given ξ. To ensure kinematic feasibility, our model does not directly regress positions. Instead, we define the generation target in the action space as $X_{0}=\left\{\left(a_{x}^{t},{\dot{\psi }}^{t}\right)|t=1,\ldots ,T_{pred}\right\}$, consisting of longitudinal acceleration $a_{x}$ and yaw rate $\dot{\psi }$. The final trajectory $\hat{Y}$ is recovered from the generated actions ${\hat{X}}_{0}$ via a kinematic integration layer, ensuring that the output inherently satisfies vehicle non-holonomic constraints.

### 2.2. Vectorized Scene Context Learning

**Figure 2:**
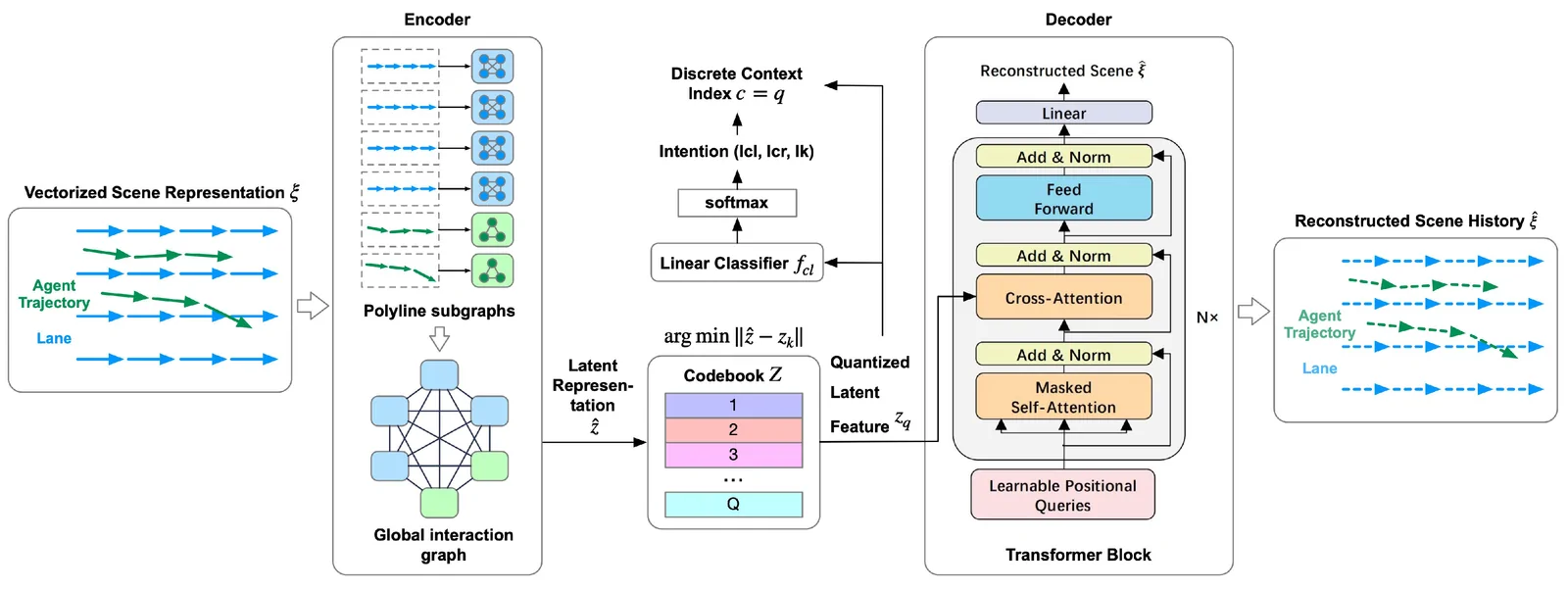
Vectorized scene context learning with VectorNet-VQ-VAE.

To effectively capture the complex interactions between agents and the road network and to categorize the infinite possibilities of traffic scenarios into discrete, semantically rich contexts, we employ a vectorized scene context learning module. As shown in Figure 2, this module integrates a VectorNet-based Encoder-Decoder architecture within a VQ-VAE framework.

#### 2.2.1. Hierarchical VectorNet Encoder

The VectorNet backbone [36] is leveraged here to encode the rich interactions among traffic participants and the surrounding road network. This hierarchical GNN operates directly on a polyline-based scene representation, thereby avoiding the information loss inherent to rasterization. Lanes and agent trajectories are approximated as ordered sequences of short vectors: map features are obtained by uniformly sampling key points along the spline of each lane and chaining them in order, while agent histories are sampled at fixed time intervals. A vector $v_{i}$ on polyline $P_{j}$ becomes a node of the graph carrying features $v_{i}=\left[d_{s},d_{e},a,j\right]$, where $d_{s}$ and $d_{e}$ are the start and end coordinates, *a* collects auxiliary attributes, and *j* identifies the parent polyline. Translational invariance is enforced by re-centering every coordinate around the target agent’s position at the last observed time step.

Based on this representation, the encoder aggregates information hierarchically, starting from the local vector level up to the global scene level. First, to exploit spatial and semantic locality, we construct subgraphs for each polyline. For a polyline $P_{j}$ containing vectors $\left\{v_{1},\ldots ,v_{L}\right\}$, a subgraph network aggregates features using a Multi-Layer Perceptron (MLP) followed by a permutation-invariant max-pooling operation $\phi_{agg}$:

(1) $$p_{j}=\phi_{agg}\left(\mathrm{MLP}(v_{i})\right),\;\forall v_{i}\in P_{j}$$

This process effectively compresses the variable-length vector sequence into a fixed-size polyline embedding $p_{j}$, encapsulating the local geometric characteristics of the road or the motion pattern of an agent.

Subsequently, to model higher-order interactions among all scene components (i.e., agents and map features), we construct a fully connected global graph where nodes are the polyline embeddings $\left\{p_{1},\ldots ,p_{M}\right\}$. Following VectorNet [36], a self-attention operation is applied to update these features by capturing long-range dependencies. Let $P\in R^{M\times d}$ denote the matrix formed by stacking all polyline embeddings, and let $P_{Q}$, $P_{K}$, and $P_{V}$ be its query, key, and value projections, respectively. The global graph update is written as:

(2) $$\hat{Z}=\mathrm{softmax}\left(\frac{P_{Q}P_{K}^{T}}{\sqrt{d_{k}}}\right)P_{V}$$

where $d_{k}$ is the dimension of the key projection. The final output corresponding to the target agent, denoted as $\hat{z}$, encapsulates the global scene context and serves as the continuous latent representation for the subsequent quantization module.

#### 2.2.2. Discrete Context Quantization

To handle the continuous, unbounded nature of interactive traffic scenarios, we introduce a discrete quantization step after global feature aggregation. As illustrated in Figure 2, the continuous global embedding $\hat{z}$ extracted by the hierarchical encoder is projected onto a finite, learnable codebook $Z=\{z_{1},...,z_{Q}\}$. Rather than employing traditional continuous latent spaces, this operation maps the current scene to the nearest semantic and geometric prototype $z_{q}$ by minimizing the Euclidean distance in the embedding space:

(3) $$z_{q}=\arg\min_{z_{k}\in Z}{∥\hat{z}-z_{k}∥}_{2}^{2}$$

Assigning the dynamic observation to a discrete context cluster c=q significantly reduces multimodal ambiguity. To ensure these discrete representations maintain both geometric fidelity and strong correlation with high-level driving maneuvers, the codebook and network parameters are optimized simultaneously through a composite objective function:

(4) $$L=L_{recon}+L_{vq}+\lambda L_{cl}$$

where the scalar coefficient λ controls the relative contribution of the intent classification objective compared to the reconstruction and quantization terms. This weighting steers the model toward semantically interpretable representations while preserving the geometric fidelity of the reconstructed scene.

The reconstruction term $L_{recon}$ quantifies the vector-wise discrepancy between the original scene input ξ and the decoded output $\hat{\xi }$, so that the discrete latent code $z_{q}$ retains the geometric information essential to the traffic scenario. It is defined as:

(5) $$L_{recon}={∥\xi -\hat{\xi }∥}_{2}^{2}$$

Simultaneously, the VQ-VAE objective $L_{vq}$ is applied to update the codebook and constrain the encoder outputs:

(6) $$L_{vq}={∥\mathrm{sg}[\hat{z}]-z_{q}∥}_{2}^{2}+\beta {∥\mathrm{sg}[z_{q}]-\hat{z}∥}_{2}^{2}$$

where sg[·] denotes the stop-gradient operation. The first term updates the codebook embeddings, while the second term (commitment loss) constrains the encoder output from fluctuating excessively.

Finally, to emphasize scenario-specific factors that influence future maneuvers, an intent classification task is incorporated into the latent space. A linear classifier $f_{cl}$ is added to predict the maneuver probability distribution *p* (i.e., LCL, LCR, or KL) based on the quantized code $z_{q}$:

(7) $$p=\mathrm{softmax}(f_{cl}\left(z_{q}\right))$$

To penalize incorrect intent predictions, a cross-entropy loss is computed against the ground truth maneuver label *s*:

(8) $$L_{cl}=-\sum s\log\left(p\right)$$

By introducing $L_{cl}$, the latent space is encouraged to form maneuver-associated codebook entries, with each cluster *q* representing a specific intersection of geometric topology and driving intent, thereby providing a structured condition for the subsequent generative stage.

Since the codebook is shaped by the supervised classification loss $L_{cl}$ with LCL, LCR, and KL labels, the learned codes are maneuver-associated scene prototypes, which are discrete summaries of the agent–map interaction context that are predictive of the labeled maneuver. Throughout this paper, the terms “intent code” and “intent prototype” are used as shorthand for these supervised maneuver-associated prototypes, consistent with the common usage of “intention” for maneuver-level classes in the trajectory prediction literature [17,18].

### 2.3. Conditioned Diffusion Model

**Figure 3:**
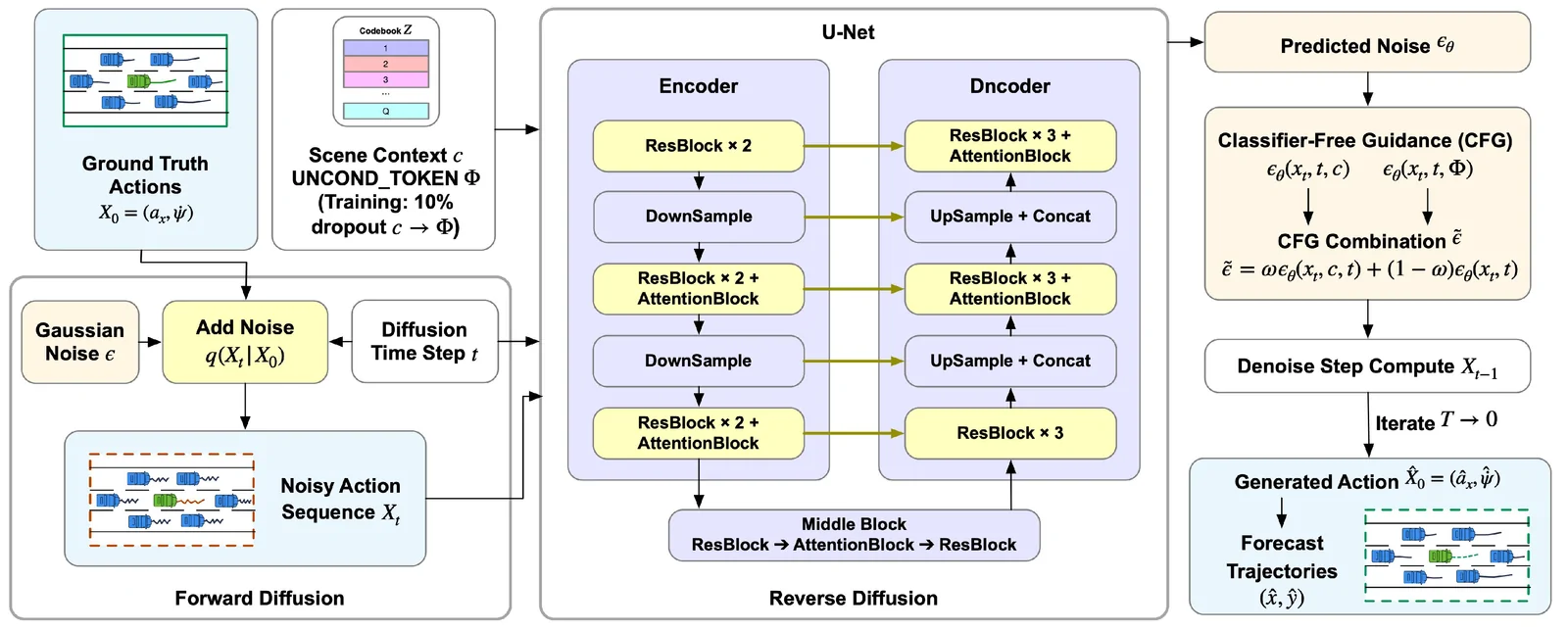
Conditional generative trajectory prediction with 2D U-Net DDPM.

As illustrated in Figure 3, the conditioned diffusion model uses a DDPM [37] to generate the future action sequence $X_{0}$, conditioned on the discrete context index *c* obtained via vectorized scene context learning.

#### 2.3.1. Denoising Diffusion Process and Network Architecture

DDPMs form the generative core of our framework, enabling the model to learn the complex conditional distribution of future actions given the context $p(X_{0}|c)$. The training process is divided into a fixed forward diffusion phase and a learnable reverse denoising phase.

In the forward phase, the initial ground-truth action sequence $X_{0}$ is systematically corrupted with Gaussian noise. This is achieved using a Markov chain that gradually adds Gaussian noise ϵ over *T* steps, following a variance schedule ${\left\{\beta_{t}\in \left(0,1\right)\right\}}_{t=1}^{T}$. The transition probability at each step is defined as $q\left(X_{t}|X_{t-1}\right)=N\left(X_{t};\sqrt{1-\beta_{t}}X_{t-1},\beta_{t}I\right)$. A key property of this process is that the state $X_{t}$ at any arbitrary timestep *t* can be sampled directly from $X_{0}$ in closed form:

(9) $$X_{t}=\sqrt{{\bar{\alpha }}_{t}}X_{0}+\sqrt{1-{\bar{\alpha }}_{t}}\epsilon ,\;\epsilon ∼N\left(0,I\right)$$

where $\alpha_{t}=1-\beta_{t}$ and ${\bar{\alpha }}_{t}=\prod_{s=1}^{t}\alpha_{s}$. As T→∞, ${\bar{\alpha }}_{t}\to 0$, ensuring that the distribution of $X_{T}$ converges to an isotropic Gaussian N(0,I).

In the reverse phase, the goal is to invert this diffusion process to recover the original data structure from pure noise. This is modeled as a parameterized Markov chain $p_{\theta }$ that learns to denoise the state $X_{t}$ in a step-by-step manner. Since our framework is condition-driven, the reverse transition is explicitly conditioned on the discrete context index *c*, derived from the vectorized scene-context learning. The transition probability is defined as a Gaussian distribution:

(10) $$p_{\theta }\left(X_{t-1}|X_{t},c\right)=N\left(X_{t-1};\mu_{\theta }\left(X_{t},t,c\right),\Sigma_{\theta }\left(X_{t},t\right)\right)$$

where $\mu_{\theta }$ and $\Sigma_{\theta }$ are the predicted mean and covariance, respectively. Following Ho et al. [37], we set the variance $\Sigma_{\theta }\left(X_{t},t\right)$ to fixed constants $\beta_{t}I$, focusing the learning capacity solely on the mean $\mu_{\theta }$.

However, instead of directly predicting the reverse-process mean $\mu_{\theta }$, it has been shown that a more stable training objective is to predict the noise component ϵ added at step *t*. By reparameterizing the Gaussian term, the mean of the reverse process can be derived from the predicted noise $\epsilon_{\theta }\left(X_{t},t,c\right)$ as follows:

(11) $$\mu_{\theta }\left(X_{t},t,c\right)=\frac{1}{\sqrt{\alpha_{t}}}\left(X_{t}-\frac{1-\alpha_{t}}{\sqrt{1-{\bar{\alpha }}_{t}}}\epsilon_{\theta }\left(X_{t},t,c\right)\right)$$

This formulation allows the model to generate new samples by sampling Gaussian noise $X_{T}$ and iteratively solving for $X_{t-1}$ using the learned noise estimator $\epsilon_{\theta }$, guided by the semantic condition *c*.

To implement this noise prediction function $\epsilon_{\theta }$, we employ a 2D U-Net architecture for the target vehicle’s action sequence. Unlike traditional sequence models that process trajectories only as 1D time series, we organize the noisy target action sequence as a compact temporal feature map of shape $\left(B,C,1,T_{pred}\right)$, where *B* is the batch size and C=2 corresponds to the action channels (longitudinal acceleration and yaw rate). Interactions with surrounding vehicles are not generated by the U-Net; instead, they are encoded beforehand by the VectorNet–VQ-VAE scene context module and injected through the discrete context index *c*. The U-Net therefore denoises the target-vehicle future while receiving interaction information through the learned condition. The discrete context index *c* is integrated via learnable embedding layers, which are added to the timestep embeddings to guide the denoising trajectory. The entire network is optimized by minimizing the simplified Mean Squared Error (MSE) between the actual noise ϵ and the predicted noise $\epsilon_{\theta }$:

(12) $$L_{simple}=E_{t,X_{0},\epsilon }\left[∥\epsilon -\epsilon_{\theta }\left(X_{t},t,c\right){∥}_{2}^{2}\right]$$

#### 2.3.2. Inference with Classifier-Free Guidance

In conditioned diffusion models, guidance involves steering the generation process by incorporating additional conditioning. We utilize Classifier-Free Guidance (CFG) [38], which enables conditional generation without an explicit external classifier. In this approach, an unconditional noise estimator $\epsilon_{\theta }\left(X_{t},t,\emptyset \right)$ and a conditional noise estimator $\epsilon_{\theta }\left(X_{t},t,c\right)$ are jointly trained within a single neural network. During training, the class identifier *c* is randomly replaced with the null token ∅ with probability p=0.1.

During the sampling process, the noise estimate ${\tilde{\epsilon }}_{\theta }$ of the guided DDPM is determined by a linear combination of the conditional and unconditional predictions:

(13) $${\tilde{\epsilon }}_{\theta }\left(X_{t},t,c\right)=\omega \epsilon_{\theta }\left(X_{t},t,c\right)+\left(1-\omega \right)\epsilon_{\theta }\left(X_{t},t,\emptyset \right)$$

where ω denotes the guidance scale. A higher ω forces the generated trajectory to strictly adhere to the identified semantic maneuver, while a lower scale preserves more diversity from the unconditional distribution. In our experiments, we set ω=2.0 for all forecast-based LCRI evaluations, which keeps the sampling protocol fixed across the trajectory and risk analyses.

#### 2.3.3. Kinematic Reconstruction

Instead of predicting positions directly, our model generates a sequence of denoised motion parameters ${\hat{X}}_{0}=\left\{\left({\hat{a}}_{x,t},{\hat{\dot{\psi }}}_{t}\right)\right\}$. The rationale behind this choice lies in the fundamental premise of diffusion models, which is to map a Gaussian noise distribution to the target data distribution. While the distribution of absolute trajectory positions is highly multi-modal and complex, the distributions of acceleration and yaw rate are naturally bounded and quasi-Gaussian. Therefore, transforming the target space into the action domain significantly simplifies both the forward noising process and the reverse learning task.

To recover the final trajectory, we employ a kinematic representation of motion subject to nonholonomic constraints. Given the predicted motion parameters, the vehicle state $s_{t}=\left[x_{t},y_{t},v_{t},\psi_{t}\right]$ is recursively updated using Euler integration. The state update logic at step t+1 is formulated as:

(14) $$\begin{array}{cc} \; & v_{t+1}=v_{t}+{\hat{a}}_{x,t}\Delta t \end{array}$$

(15) $$\begin{array}{cc} \; & \psi_{t+1}=\psi_{t}+{\hat{\dot{\psi }}}_{t}\Delta t \end{array}$$

(16) $$\begin{array}{cc} \; & x_{t+1}=x_{t}+v_{t+1}\cos\left(\psi_{t+1}\right)\Delta t \end{array}$$

(17) $$\begin{array}{cc} \; & y_{t+1}=y_{t}+v_{t+1}\sin\left(\psi_{t+1}\right)\Delta t \end{array}$$

where Δt is the time interval. This explicit integration ensures that the predicted paths satisfy vehicle kinematic constraints, preventing the unrealistic drift in position- based predictions.

The integration guarantees that position, speed, and heading evolve consistently under the nonholonomic vehicle model. Two mechanisms keep the generated motions within a realistic dynamic envelope. First, the action space is bounded by construction. The denoised actions are clipped to the normalized range before integration, which caps the longitudinal acceleration at $|{\hat{a}}_{x}|\;\leq 1.5\;m/s^{2}$ and the yaw rate at $|\hat{\dot{\psi }}|\leq 0.05$ rad/s, both well inside the physical capability of passenger cars and consistent with the action ranges observed in the highD training data. Second, we verified the dynamic envelope empirically on trajectories generated under the evaluation protocol, 2000 samples over 200 test conditions, K=10, ω=2.0. The 99th percentiles of the generated absolute longitudinal acceleration, lateral acceleration $v\hat{\dot{\psi }}$, and jerk are $1.41\;m/s^{2}$, $0.44\;m/s^{2}$, and $0.55\;m/s^{3}$, respectively, all within commonly used comfort envelopes for highway driving and of the same order as the corresponding ground truth statistics. Road geometry is not imposed as a hard constraint at the integration stage. It enters through the discrete scene context code, which conditions the generator on the vectorized lane layout.

### 2.4. Predictive Lane-Change Risk Assessment

The central goal of this work is to translate the target agent’s multimodal forecasts into a proactive, interpretable measure of lane-change risk. To this end, we couple the generated trajectories with a rear-end collision risk model and aggregate the per-interaction risk into a single Lane-Change Risk Index (LCRI) [11] over the prediction horizon. A lane change is risky primarily because of its longitudinal interaction with the four key vehicles that bound the maneuver: the preceding and following vehicles in the current lane, and the preceding and following vehicles in the target lane. The future trajectory of the target agent is the multimodal forecast ${\left\{{\hat{Y}}_{k}\right\}}_{k=1}^{K}$ produced by the conditioned diffusion model. Each surrounding vehicle is propagated using a constant-velocity model from its last observed state, yielding a smooth, physically plausible future and keeping the entire pipeline dependent only on quantities available at prediction time. Although with this simplification, the reactive braking of a following vehicle, the reactive behavior most relevant to rear-end risk, is not ignored. We model it stochastically in the Monte Carlo risk computation described below, where each trial samples a reaction time and braking deceleration for the follower. Furthermore, we empirically bound the impact of this simplification in our evaluation. The realized-future reference LCRI in Section 4.5 is computed on the actual recorded trajectories of all vehicles, including whatever reactive behavior the surrounding drivers in fact performed, so the agreement reported there already includes the full effect of the constant-velocity assumption over the prediction horizon.

At each predicted timestep, the risk between the target agent and one key vehicle is quantified by a rear-end collision risk that jointly accounts for collision likelihood and collision severity, following the surrogate-safety formulation of Shangguan et al. [8,11]. For a leader–follower pair with relative gap, leader speed, and follower speed read off the forecast at that frame, a Monte Carlo simulation samples the lead disturbance deceleration, the follower reaction time, and the follower maximum braking deceleration from their empirical distributions, and resolves four rear-end scenarios in closed form to decide whether a collision occurs and, if so, its severity the square of the absolute speed difference (SASD) between the two vehicles at the moment of impact. The three stochastic factors follow the empirically calibrated distributions adopted in Shangguan et al. [8]. The disturbance deceleration imposed on the lead vehicle follows a shifted gamma distribution with shape 17.315, scale 0.128, and shift 0.657 (in $m/s^{2}$), originally calibrated on naturalistic rear-end conflict data by Kuang et al. [39]. The follower reaction time follows a lognormal distribution with parameters (0.17,0.44) plus a fixed braking coordination time of 0.175 s. The maximum available deceleration rate of the follower follows a truncated normal distribution with mean $8.45\;m/s^{2}$ and standard deviation $1.4\;m/s^{2}$, truncated to $\left[4.23,12.68\right]\;m/s^{2}$. The rear-end collision risk index for key vehicle *i* at the considered frame is:

(18) $${\mathrm{RCRI}}_{i}=\frac{1}{N_{mc}}\sum_{j=1}^{N_{mc}}{\mathrm{crash}}_{ij}\cdot {\tilde{\mathrm{SASD}}}_{ij},$$

where $N_{mc}$ is the number of Monte Carlo trials, ${\mathrm{crash}}_{ij}\in \left\{0,1\right\}$ indicates whether trial *j* results in a rear-end collision, and ${\tilde{\mathrm{SASD}}}_{ij}$ is the normalized severity.

The severity is normalized by a fixed constant, ${\tilde{\mathrm{SASD}}}_{ij}={\mathrm{SASD}}_{ij}/{\mathrm{SASD}}_{\max}$ with ${\mathrm{SASD}}_{\max}=130\;m^{2}/s^{2}$, and the resulting risk index is clipped to [0,1]. The constant was calibrated so that our vectorized implementation reproduces the RCRI values reported for the reference car-following episode in Shangguan et al. [8] to within 0.01, thereby keeping the index numerically comparable to the original formulation. The identical normalization is applied to the forecast-based and the realized-future evaluations, so it does not affect their comparison. Because the model evaluates the longitudinal closing process under stochastic but realistic braking behavior, it assigns a continuous, non-zero risk to close-but-not-colliding interactions that a binary distance threshold would miss. This is precisely the property required on naturalistic data, where almost no trajectory ends in an actual collision.

The four per-vehicle risks are fused over the maneuver into a single index through a fault-tree formulation, in which the lane change is treated as a system whose failure corresponds to an unsafe interaction with any of the four key vehicles:

(19) $$\mathrm{LCRI}=1-\prod_{i=1}^{4}\left(1-{\mathrm{RCRI}}_{i}\right),$$

where each ${\mathrm{RCRI}}_{i}$ is taken as the maximum over the prediction horizon for that vehicle. Surrounding positions that are absent from the scene contribute ${\mathrm{RCRI}}_{i}=0$, thereby leaving the product unchanged. RCRI models the longitudinal closing process between a leader–follower pair, so the LCRI quantifies the rear-end conflict potential of the lane change with respect to its four bounding vehicles. Lateral and sideswipe conflicts—for example, a collision with a vehicle alongside during the lateral movement itself—are not explicitly modeled, although they are partially reflected in the index because an alongside vehicle becomes a leader or follower in the target lane as the maneuver progresses. Accordingly, LCRI should be read throughout this paper as a rear-end-based lane-change risk index. Following the three-level operational grouping in [11], we stratify LCRI into low-risk, medium-risk, and high-risk levels. Because the target-agent term of every pair is drawn from the diffusion forecast, the *K* samples yield a distribution of LCRI values. We report the sample mean as the expected risk and retain the per-sample maximum as a worst-case diagnostic.

Crucially, every prediction is produced under a discrete intent code *c* (Section 2), and the LCRI is computed from the same forward pass. The risk index can therefore be grouped by code, allowing us to analyze the association between learned discrete prototypes and lane-change risk. We analyze this relationship quantitatively in Section 4.

## 3. Experiment Settings

### 3.1. Data Preparation

We evaluate our framework on the highD dataset [40], a large-scale naturalistic vehicle trajectory dataset recorded by drone-mounted cameras over German highways. Unlike traditional datasets collected via vehicle-mounted sensors, highD provides highly accurate positioning with an error of less than 10 cm from an aerial bird’s-eye view. The dataset aggregates data from roughly 110,500 vehicles and approximately 45,000 km of cumulative driving distance across six recording sites, covering a wide range of traffic densities and interaction patterns.

The raw trajectory data is sampled at 25 Hz. For each scenario, we extract a historical observation window of $T_{obs}=3$ s (75 frames) and predict the future trajectory for $T_{pred}=5$ s (125 frames). To capture local interactions, we construct a heterogeneous interaction graph for each target agent. Specifically, we select up to N=8 spatial neighbors based on their relative positions (i.e., preceding, following, and alongside vehicles on the current and adjacent lanes). The coordinates of all agents and vectorized lane boundaries are normalized to the target agent’s frame at the last observed timestep (t=0).

A significant challenge in naturalistic driving datasets is the extreme class imbalance, where KL maneuvers vastly outnumber LC maneuvers. To prevent the model from collapsing into a trivial solution that predicts only straight paths, we apply an undersampling strategy during training-time preprocessing: all LCL and LCR samples are retained, while KL samples are randomly downsampled. We use files 01–50 for training and files 51–60 for testing. The split is performed at the recording level. Every vehicle appears in exactly one highD recording, thus no trajectory windows extracted from the same vehicle can appear in both the training and the test subsets. Within each recording, samples are extracted from every eligible vehicle track with a sliding window at a stride of 10 frames (0.4 s). Consecutive windows from the same vehicle therefore overlap, but only within the same subset. This overlap enlarges the effective sample count without introducing any train–test contamination. The balanced training split contains 117,658 samples, including 42,015 KL, 40,308 LCR, and 35,335 LCL samples. For evaluation under the natural driving distribution, we use the unbalanced test split with 255,069 samples, including 238,687 KL, 8609 LCR, and 7773 LCL samples.

### 3.2. Implementation Details

The encoder backbone is implemented as a hierarchical VectorNet. Each subgraph branch uses a 3-layer MLP, while the global interaction graph adopts self-attention with 4 attention heads and a hidden size of 128. The codebook is configured with Q=60 entries and an embedding dimension of D=36, and the commitment loss weight is fixed at β=0.25. Training is performed for 100 epochs with a batch size of 64 and the Adam optimizer at a learning rate of $4.5\times 10^{-6}$. To let the model first focus on geometric reconstruction, a dynamic schedule is applied to the intent classification weight λ. A 10-epoch warm-up with λ=0 is followed by a 20-epoch linear ramp-up that brings λ to 1.0.

The generative model is a 2D U-Net conditioned on discrete context indices extracted from vectorized scene-context learning. Since the diffusion model generates only the target vehicle’s future action sequence, the noisy action input is organized as a 4D tensor with shape $\left(B,C,H,W\right)=\left(B,C,1,T_{pred}\right)$. Here, B=64 is the batch size, C=2 corresponds to the action channels, and the singleton height dimension allows the temporal action sequence to be processed by 2D convolutional blocks. Inter-agent information enters the diffusion model through the discrete scene context code rather than through generated multi-agent action channels. The network consists of residual blocks with channel multipliers of (1,2,4), with attention blocks applied at selected downsampled temporal resolutions. We employ a cosine noise schedule with T=1000 diffusion steps. The model is optimized to minimize the Mean Squared Error (MSE) of the noise prediction. During training, the condition *c* is dropped with probability p=0.1. During inference, we generate K=10 samples per scenario with guidance scale ω=2.0. The predicted actions are integrated by the kinematic layer with Δt=0.04 s.

All models were implemented using the PyTorch 2.3.0 framework. The training and inference experiments were conducted on a single NVIDIA 2080 Ti GPU with 11 GB of RAM. The training-stage hyperparameters were selected as follows. The commitment weight β=0.25 follows the standard VQ-VAE setting [41], and the training is not sensitive to it in our experiments. The codebook size Q=60 was chosen to provide sufficient capacity for intra-maneuver variation while keeping each code supported by enough training samples. A larger codebook produces finer-grained prototypes but increases the number of rarely used codes and weakens per-code statistics, whereas a smaller codebook forces distinct interaction patterns to share a single code and reduces the semantic purity of the prototypes. The warm-up schedule of the intent classification weight λ allows the encoder to first learn geometric reconstruction before the semantic objective is introduced. Activating λ too early or with a larger final value causes the codebook to collapse onto the three maneuver labels, at the cost of geometric fidelity. On the other hand, a smaller final value weakens the association between codes and maneuvers. The number of Monte Carlo trials $N_{mc}=2000$ was chosen such that the standard error of the RCRI estimate is below the spacing of the three-level risk thresholds. The learning rate and the number of epochs were selected based on convergence of the validation loss. The inference-stage parameters ω and *K* are analyzed quantitatively in Section 4.4. For ease of reference, Table 1 summarizes the main symbols and tunable parameters of the proposed framework together with the values used in the experiments.

### 3.3. Baseline Models

To evaluate the effectiveness of the proposed framework, we compare it with several state-of-the-art trajectory prediction models reported in the literature. These baselines cover a range of methodologies, including recurrent neural networks, transformer-based architectures, and recent diffusion probabilistic models. The baseline results for model comparisons are taken from the original studies, which were evaluated on the highD dataset.

- S-LSTM [42]: An early baseline that pairs LSTM cells for individual motion modeling with a social pooling layer for aggregating features from neighboring agents; it was first proposed for pedestrian trajectory forecasting.
- CS-LSTM [20]: A vehicle-oriented extension of S-LSTM that replaces the social pooling layer with a convolutional one to better preserve the spatial grid surrounding the ego vehicle.
- MHA-LSTM (+f) [21]: A multi-head attention model that scores the relative importance of surrounding vehicles. By combining interaction features with historical motion, it explicitly weights the influence of neighbors during forecasting.
- iNATran (M) [24]: An intention-aware, non-autoregressive Transformer that combines social and temporal attention to model spatiotemporal interactions and uses an intent-driven query design to generate multimodal futures in parallel.
- DACR-AMTP [16]: An adaptive multi-modal predictor that incorporates a collision-risk-driven drivable area into a graph-augmented multi-head attention pipeline, foregrounding safety and risk in interactive scenes.

### 3.4. Evaluation Metrics

We utilize three standard displacement metrics: Root Mean Square Error (RMSE), Average Displacement Error (ADE), and Final Displacement Error (FDE). Given the multimodal nature of our predictions, these metrics are computed using the best-matching sample among the *K* generated trajectories for each test case. RMSE assesses temporal accuracy at specific time horizons throughout the prediction window. ADE calculates the mean Euclidean distance over all predicted timesteps, whereas FDE isolates the endpoint error at the final prediction frame $T_{pred}$. They are calculated as follows:

(20) $${\mathrm{RMSE}}_{t}=\sqrt{\frac{1}{M}\sum_{m=1}^{M}\min_{k\in \{1,\ldots ,K\}}\left({\left({\hat{x}}_{t}^{\left(m,k\right)}-x_{t}^{\left(m\right)}\right)}^{2}+{\left({\hat{y}}_{t}^{\left(m,k\right)}-y_{t}^{\left(m\right)}\right)}^{2}\right)}$$

(21) $$\mathrm{ADE}=\frac{1}{M}\sum_{m=1}^{M}\min_{k\in \{1,\ldots ,K\}}\left(\frac{1}{T_{\mathrm{pred}}}\sum_{t=1}^{T_{\mathrm{pred}}}\sqrt{{\left({\hat{x}}_{t}^{\left(m,k\right)}-x_{t}^{\left(m\right)}\right)}^{2}+{\left({\hat{y}}_{t}^{\left(m,k\right)}-y_{t}^{\left(m\right)}\right)}^{2}}\right)$$

(22) $$\mathrm{FDE}=\frac{1}{M}\sum_{m=1}^{M}\min_{k\in \{1,\ldots ,K\}}\sqrt{{\left({\hat{x}}_{T_{\mathrm{pred}}}^{\left(m,k\right)}-x_{T_{\mathrm{pred}}}^{\left(m\right)}\right)}^{2}+{\left({\hat{y}}_{T_{\mathrm{pred}}}^{\left(m,k\right)}-y_{T_{\mathrm{pred}}}^{\left(m\right)}\right)}^{2}}$$

where $\left({\hat{x}}_{t}^{\left(m,k\right)},{\hat{y}}_{t}^{\left(m,k\right)}\right)$ represents the *k*-th predicted trajectory sample for the *m*-th test case, $\left(x_{t}^{\left(m\right)},y_{t}^{\left(m\right)}\right)$ represents the corresponding ground-truth coordinate at prediction timestamp *t*, and *M* is the total number of test samples. We report RMSE at specific horizons (i.e., 1 s, 2 s, 3 s, 4 s, 5 s) to analyze temporal accuracy. As a complement to the best-of-*K* protocol, single-sample results (K=1) are reported in the sensitivity analysis of Section 4.4.

For the risk assessment, we evaluate both continuous and ordinal agreement. Continuous agreement is measured by Pearson correlation and Spearman rank correlation between the forecast-based LCRI and the LCRI computed from realized future trajectories. Ordinal agreement is evaluated using the three-level LCRI classes following [11]: low risk (LCRI≤0.03), medium risk (0.03<LCRI≤0.30), and high risk (LCRI>0.30). We report the three-class confusion matrix, overall accuracy, weighted-F1, and high-risk recall.

## 4. Experiment Results

### 4.1. Analysis of Discrete Context Latent Space

The effectiveness of vectorized scene context learning is first evaluated by analyzing how complex traffic scenarios are disentangled into semantically meaningful latent codes. The VQ-VAE module is designed to discretize the infinite continuous scenario space into a finite set of learnable codebook entries $Z=\{z_{1},\ldots ,z_{Q}\}$. Each discrete code $z_{q}$ represents a specific geometric configuration and corresponds to a distinct driving maneuver (i.e., LCL, KL, or LCR), thereby reducing ambiguity for the downstream diffusion generator.

**Figure 4:**
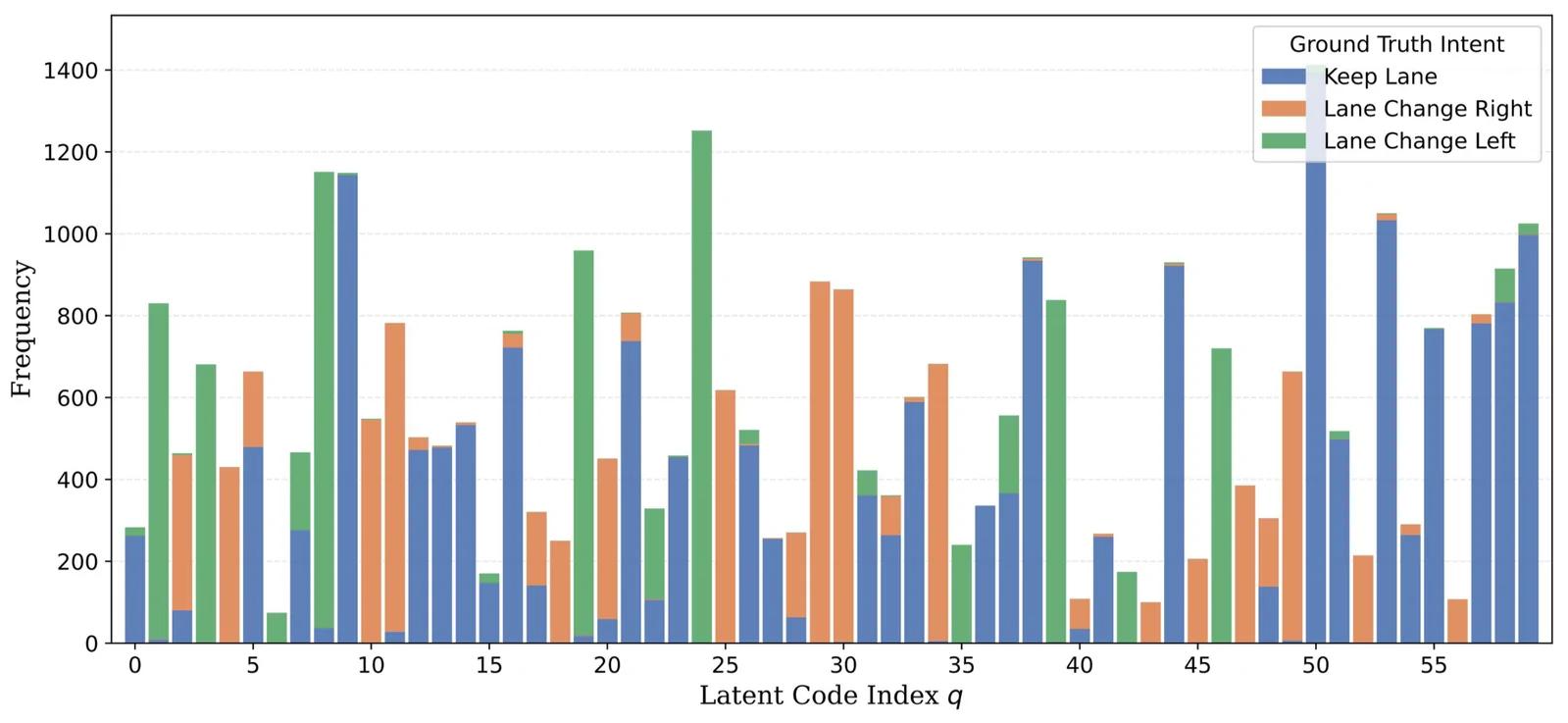
Distribution of latent codes by driving intent.

Figure 4 presents the stacked histogram of the latent code usage. The horizontal axis represents the codebook indices q∈{0,…,59}, and the vertical axis shows the frequency with which each code is selected by the encoder. The color of each bar segment corresponds to the ground-truth maneuver class of the target agent: blue for lane-keeping, orange for right lane-changing, and green for left lane-changing. As observed in Figure 4, most active codebook entries are dominated by a single color segment. For instance, specific indices are triggered almost exclusively by left lane-changing maneuvers (green bars), while others correspond to lane-keeping behaviors (blue bars). This strong correlation indicates that the auxiliary intent classification loss $L_{cl}$ effectively regularized the latent space, encouraging the model to disentangle different driving modes into separate clusters. Furthermore, the histogram also reveals the usage pattern of the codebook. While some codes are frequently used to capture common driving scenarios, others occur infrequently, suggesting that the model effectively compressed the scenario space into a subset of active prototypes.

Some codes exhibit mixed color segments—for example, bars containing both blue and orange portions. This indicates a degree of ambiguity, in which geometrically similar scenarios could yield different future outcomes. Nevertheless, the overall high purity of the bars suggests that the discrete context *c* provides a reliable, low-entropy condition for the subsequent diffusion generation stage, thereby reducing modality confusion.

To quantify these observations, we characterize the learned discrete latent space with information-theoretic measures, treating the codebook index *C* and the ground-truth maneuver class *M* as random variables. Table 2 summarizes the results. The marginal codebook entropy is H(C)=3.74, against the uniform upper bound of ln60=4.09, corresponding to a codebook perplexity of $e^{H(C)}\approx 42.1$ effective codes out of 60. The codebook therefore operates at roughly 91% of its capacity: the encoder spreads probability mass over most of the codebook rather than collapsing onto a few entries, while reserving a subset of codes for rare interaction patterns. Cluster purity, defined per code as the fraction of its samples belonging to its majority maneuver class, averages 0.83 over the 59 codes with at least five test samples, and 27 of these codes reach a purity of at least 0.90. The impure codes correspond to the mixed bars in Figure 4. The mutual information between codes and maneuvers is I(C;M)=0.63, which accounts for 58% of the maneuver entropy H(M)=1.09; the discrete code thus carries substantial maneuver-discriminative information, consistent with the visual purity of the histogram in Figure 4. At the same time, the conditional entropy H(M∣C)=0.47 shows that the code does not fully determine the maneuver. This residual uncertainty is consistent with the design of the framework. The code summarizes the scene-level interaction context, while the diffusion stage accounts for the remaining within-code variability in future motion. From an information-theoretic perspective, the VQ-VAE acts as a lossy compressor of the traffic scene, preserving maneuver-relevant information in a discrete bottleneck.

### 4.2. Quantitative Results

Table 3 summarizes the quantitative performance of the proposed framework against the baseline models on the highD dataset. The evaluation metrics include RMSE over a 5-s prediction horizon, as well as ADE and FDE@5s. IntentDiff achieves competitive trajectory prediction accuracy, with the best ADE (0.42 m) and FDE (1.11 m) among models for which these metrics are available, and the best or tied-best average RMSE (0.39 m). Compared with traditional RNN-based methods, S-LSTM [42] and CS-LSTM [20], IntentDiff reduces the average RMSE by approximately 74%, indicating the advantage of the proposed generative formulation in this benchmark.

Analyzing the RMSE variation over the prediction horizon reveals the temporal robustness of our model. While the Transformer-based iNATran(M) [24] exhibits a slight advantage in the short-term horizon (1 s and 2 s), the proposed framework surpasses it in the mid-term (3 s and 4 s), achieving the lowest errors of 0.18 m and 0.52 m, respectively. Although DACR-AMTP [16] reports the lowest RMSE at the 5-s mark (1.01 m), the proposed framework remains highly competitive (1.05 m) and obtains a lower reported FDE (1.11 m vs. 1.69 m). This suggests that the proposed framework can maintain globally consistent target-vehicle trajectories while preserving competitive pointwise accuracy.

### 4.3. Qualitative Results

**Figure 5:**
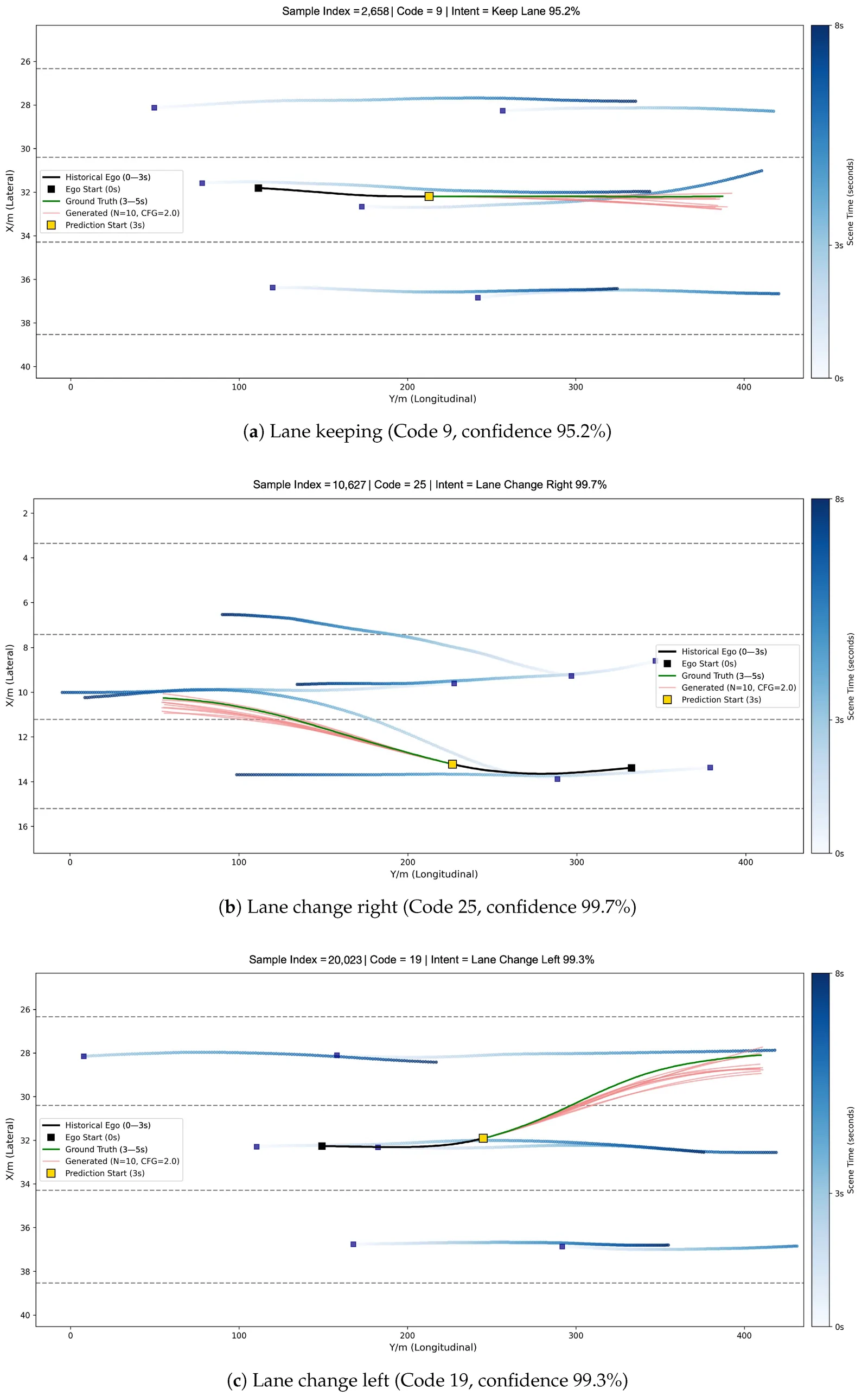
Trajectory prediction results in explicit-intent scenarios.

**Figure 6:**
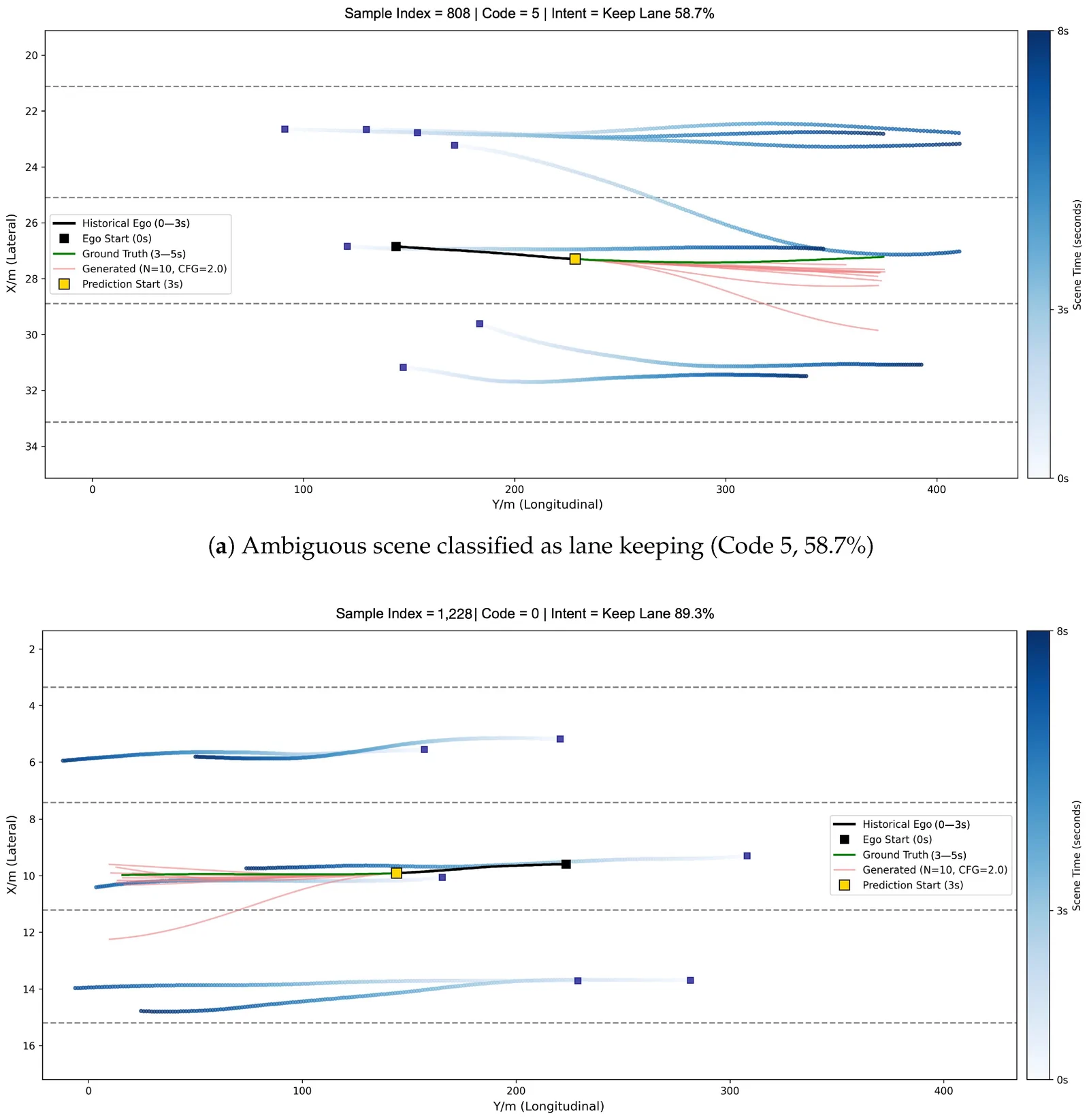
Trajectory prediction results in ambiguous scenarios.

To visually evaluate the proposed framework’s generation capabilities in different traffic scenarios, Figure 5 and Figure 6 show the prediction results for five representative test samples, grouped into explicit-intent scenarios and ambiguous scenarios. In the visualization, the black solid line represents the historical observation trajectory of the target vehicle over the past 3 s, the green solid line represents the ground truth trajectory for the next 5 s, and the pink cluster of lines shows the 10 predicted trajectories generated by the model under CFG ω=2.0. The yellow square marks the start time of the prediction.

Figure 5a, 5b and 5c illustrate typical lane-keeping, lane-changing right, and lane-changing left scenarios, respectively. In these cases, the proposed vectorized scene context learning module demonstrates high intent recognition accuracy. In Figure 5a, the model correctly identified the lane-keeping intention (Code 9) with a probability of 95.2%. The generated trajectory bundle converges tightly to the lane centerline and highly overlaps with the real trajectory, illustrating the model’s accuracy in steady-state driving scenarios. In Figure 5b and 5c, the model predicted lane-changing behavior with high confidence levels of 99.7% and 99.3%, respectively. The results show that the generated lane-changing trajectory not only matched the ground-truth value at the endpoint but also exhibited natural, kinematically consistent characteristics in movement timing and curvature. These examples suggest that the conditioned diffusion model can generate smooth and kinematically consistent lane-changing trajectories under the guidance of discrete intentions.

Figure 6a,b show two more complex interaction scenarios (Sample Index 808 and 1228). Unlike the high confidence levels in the previous cases, the model’s prediction probabilities for lane-keeping intent in these two scenarios are 58.7% and 89.3%. This lower confidence level typically reflects the ambiguity of the scenario itself, such as when the vehicle is at the edge of the lane or exhibits a potential tendency to change lanes. Visualization shows that the generated trajectory clusters exhibit greater lateral dispersion compared to Figure 5a. This diversity indicates the proposed framework’s ability to capture aleatoric uncertainty in the data, avoiding collapse into a single pattern when the scenario intent is unclear and instead generating a distribution that covers multiple possibilities, thereby supporting robust predictions.

In summary, the qualitative results indicate that the proposed framework can flexibly adjust its generation strategy based on the discrete context code extracted from the scenario. In explicit scenarios (e.g., Figure 5a–c), the model exhibits strong constraints and high accuracy; in ambiguous scenarios (e.g., Figure 6a,b), the model demonstrates reasonable diversity. Furthermore, the generated trajectories maintain good smoothness, consistent with the intended effect of introducing a kinematic integral layer during the diffusion process.

### 4.4. Ablation Study

The two inference-stage parameters, the guidance scale ω and the number of generated samples *K*, directly control the trade-off between intent adherence, diversity, and computational cost. This subsection analyzes their effects on prediction performance and explains the experimental settings.

The influence of the classifier-free guidance scale ω is investigated first. As summarized in Table 4, the prediction performance exhibits a distinct U-shaped trend with respect to ω. With ω=0, the guided noise estimate reduces to the unconditional branch learned through condition dropout during training, so the sampler draws from an unconditional diffusion model and the discrete intent code plays no role in generation. In the absence of guidance (ω=0), the model fails to strictly adhere to the target intent, resulting in a high displacement error (ADE 1.15 m). This gap isolates the contribution of intent conditioning as such, under otherwise identical model, training corpus, and sampling protocol conditions. Whether the condition must be discrete is a separate question, which is examined by the discrete–continuous conditioning ablation at the end of this subsection. Increasing ω to 2 improves accuracy by effectively directing the diffusion process toward the conditioned maneuver. However, overly strong guidance (ω=5) degrades performance (ADE increases to 0.75 m) by restricting the diversity of the generated distribution and may lead to unnatural, over-constrained trajectories. For risk assessment, this loss of diversity is particularly undesirable because an over-concentrated forecast distribution underestimates the uncertainty in future motion. Consequently, ω=2 is chosen for all experiments.

Since the proposed framework is probabilistic, the number of sampled trajectories *K* during inference directly affects the coverage of potential futures. We investigate the performance trade-off by varying K∈{1,5,10,15,20}, as shown in Table 5. When K=1, the model performance is limited (ADE 0.98 m) due to the stochastic nature of sampling. As *K* increases to 10, both ADE and FDE improve significantly to 0.42 m and 1.11 m, respectively, indicating that the model effectively covers the ground-truth mode. Increasing *K* from 1 to 10 incurs only a marginal increase in latency (from 32 ms to 45 ms), whereas further increasing *K* to 20 yields diminishing returns in accuracy, as ADE decreases by only 0.02 m, while raising the inference time to 68 ms. We therefore adopt K=10, which balances mode coverage against computational cost.

The framework’s computational profile is as follows. The VectorNet–VQ-VAE scene encoder has 1.49 million parameters, and the diffusion U-Net has 15.13 million parameters, for a total of approximately 16.6 million parameters. The inference times reported in Table 5 are end-to-end per scenario. They cover scene encoding and quantization, conditional denoising with classifier-free guidance for all *K* samples, kinematic integration, and the Monte Carlo LCRI computation. The cost is dominated by the iterative denoising of the diffusion model; because the *K* samples are generated in a single batched pass, the latency grows sublinearly with *K* (from 32 ms at K=1 to 68 ms at K=20). The Monte Carlo risk stage is fully vectorized over the four key vehicles, the prediction horizon, and the $N_{mc}=2000$ trials, and evaluates the four rear-end scenarios in closed form without any per-trial simulation loop, so it contributes only a small fraction of the total latency. A distance prefilter further skips pairs whose gap never falls below 60 m. At the adopted setting (K=10, ω=2.0), the complete pipeline processes a scenario in 45 ms, corresponding to an update rate above 20 Hz, which matches the replanning frequencies typically used in proactive risk monitoring.

Furthermore, to isolate the contribution of the discrete bottleneck at the diffusion conditioning interface, we perform an ablation that replaces the integer intent code c∈{0,…,59} with the pre-quantization continuous embedding $z_{e}\in R^{36}$ produced by the Stage 1 encoder. The same Stage 1 weights are reused. Stage 2 is retrained with an identical training configuration (CFG condition-drop probability *p* = 0.1), and the inference protocol is unchanged (*K* = 10, ω = 2.0). In the continuous variant, the Embedding(Q+1) layer in the U-Net is replaced by a two-layer MLP that maps $R^{36}\to R^{\mathrm{time}\_\mathrm{embed}\_\dim}$. Classifier-free guidance is implemented by zeroing $z_{e}$ with probability *p* = 0.1 during training, in place of the integer unconditional token used in the discrete variant. At inference, the same continuous $z_{e}$ is fed to the U-Net, and CFG uses the zero vector as the unconditioning signal.

Table 6 compares the two variants on the highD test set under the locked evaluation protocol. The two variants are nearly indistinguishable. The continuous variant is marginally better at the 2 s and 3 s horizons and in FDE, whereas the discrete variant is marginally better at the 4 s and 5 s horizons and in ADE, with an identical average RMSE of 0.39 m. The differences are small throughout, at most 0.02 m in RMSE and 0.06 m in FDE, so we consider Table 6 to be near-parity. The ablation therefore shows that quantizing the condition costs essentially no prediction accuracy, even though the 36-dimensional $z_{e}$ retains strictly more information than a single integer code. Moreover, because the continuous variant bypasses the quantizer and conditions the diffusion model directly on the unquantized encoder output, this comparison also captures the downstream effect of VQ quantization at the generation interface. The discrete bottleneck is therefore not an accuracy-motivated design choice. Its role in the framework is to provide interpretable intent prototypes, the code-by-code risk stratification, and the information-theoretic characterization of the learned representation.

### 4.5. Predictive Lane-Change Risk Assessment Results

We evaluate the proposed framework as a forecast-based estimator of proactive lane-change risk. The target-agent trajectory is generated by the intent-conditioned diffusion model under CFG ω=2.0 with K=10 samples. The four surrounding vehicles are modeled using a constant-velocity model, and the rear-end collision risk for each vehicle is estimated using $N_{mc}=2000$ Monte Carlo trials. Both the forecast-based LCRI and the reference LCRI are computed using the same surrogate-safety formulation as in [8,11]. They differ only in the trajectories on which the index is evaluated. This involves comparing predicted futures available at decision time versus the futures that are actually realized in the data. By holding the risk formula fixed, we isolate the quantity under test. The agreement between the two sides measures how well the predicted trajectories preserve the future conflict structure of the scene. This includes gaps, closing speeds, and their timing, which is the contribution of the forecasting framework. It serves as a consistency check against a retrospective surrogate index rather than a validation against actual collision outcomes. The connection between the LCRI formulation itself and real crash risk relies on the empirical grounding of the underlying surrogate measure and is not re-established here.

We first examine whether LCRI computed from predicted futures is consistent with LCRI computed from realized futures. A total of 1500 lane-change samples are drawn from the natural-distribution test set, including 750 LCR and 750 LCL maneuvers. For each sample, LCRI is computed once from the forecast available at decision time and once from the realized future trajectories of the target vehicle and its neighbors. Table 7 reports the agreement between the forecast-based and realized-future LCRI values.

The forecast-based LCRI shows strong consistency with the realized-future LCRI, as shown in Table 7. It obtains a Pearson correlation of 0.886 and a Spearman correlation of 0.875, indicating that the predicted risk values preserve both linear agreement and rank order. Under the three-level risk thresholds following [11], the forecast-based assessment achieves an accuracy of 0.874 and a weighted F1 score of 0.878. The high-risk recall is 0.863, indicating that most high-risk lane changes, as identified by the realized-future surrogate index, are identified before the maneuver is completed. This agreement also quantifies the practical impact of the constant-velocity neighbor model discussed in Section 2.4. The reference index is computed on the actual recorded trajectories of all vehicles, including the reactive braking, yielding, or acceleration that surrounding drivers actually performed, whereas the forecast-based index propagates neighbors at constant velocity. The discrepancy introduced by ignoring this reactive feedback is therefore contained in the reported correlations and confusion matrix. On highD highway traffic, the constant-velocity simplification does not systematically distort the risk ranking over the 5-s horizon.

To quantify the dataset-level statistical reliability of the four aggregate agreement metrics in Table 7, we compute 95% normal-approximation bootstrap confidence intervals (point estimate ± 1.96 bootstrap standard errors) by resampling the 1500 evaluated scenarios 1000 times. As shown in Table 8, the narrow intervals confirm that the reported agreement is robust to scenario sampling. Even at the lower endpoints of these intervals, all four metrics remain above 0.85, so the qualitative conclusion that the forecast-based assessment reproduces the realized-future ordering is not sensitive to which scenarios are included.

To quantify inference-time stochasticity, we further evaluated the LCRI consistency metrics under *N* = 5 diffusion sampling seeds, with the scenario-subsampling seed held fixed to isolate inference randomness from scenario selection. The standard deviation across the five inference seeds was 0.003 for Pearson, 0.003 for Spearman, 0.005 for accuracy, 0.005 for weighted F1, and 0.006 for high-risk recall, as shown in Table 9. These seed-to-seed standard deviations are small relative to the bootstrap CI widths in Table 8 (0.031–0.064), indicating that the reported aggregate metrics are dominated by dataset-level variation rather than by sampling stochasticity at inference time. The point estimates in Table 7 correspond to a single sampling seed, and they deviate from the five-seed means by at most 0.007, i.e., within about twice the seed-level standard deviations.

**Figure 7:**
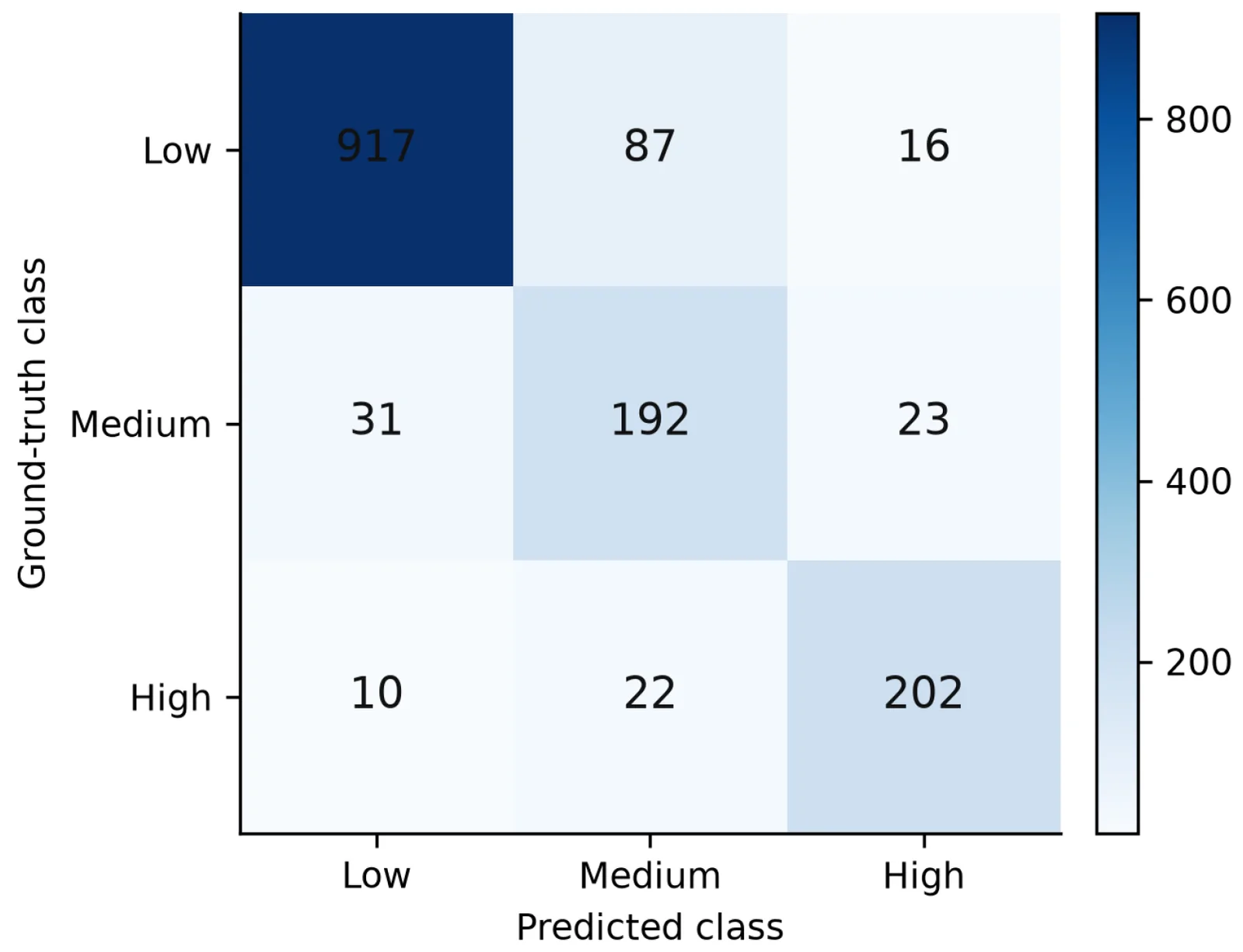
Confusion matrix for three-level forecast-based LCRI risk classification.

**Figure 8:**
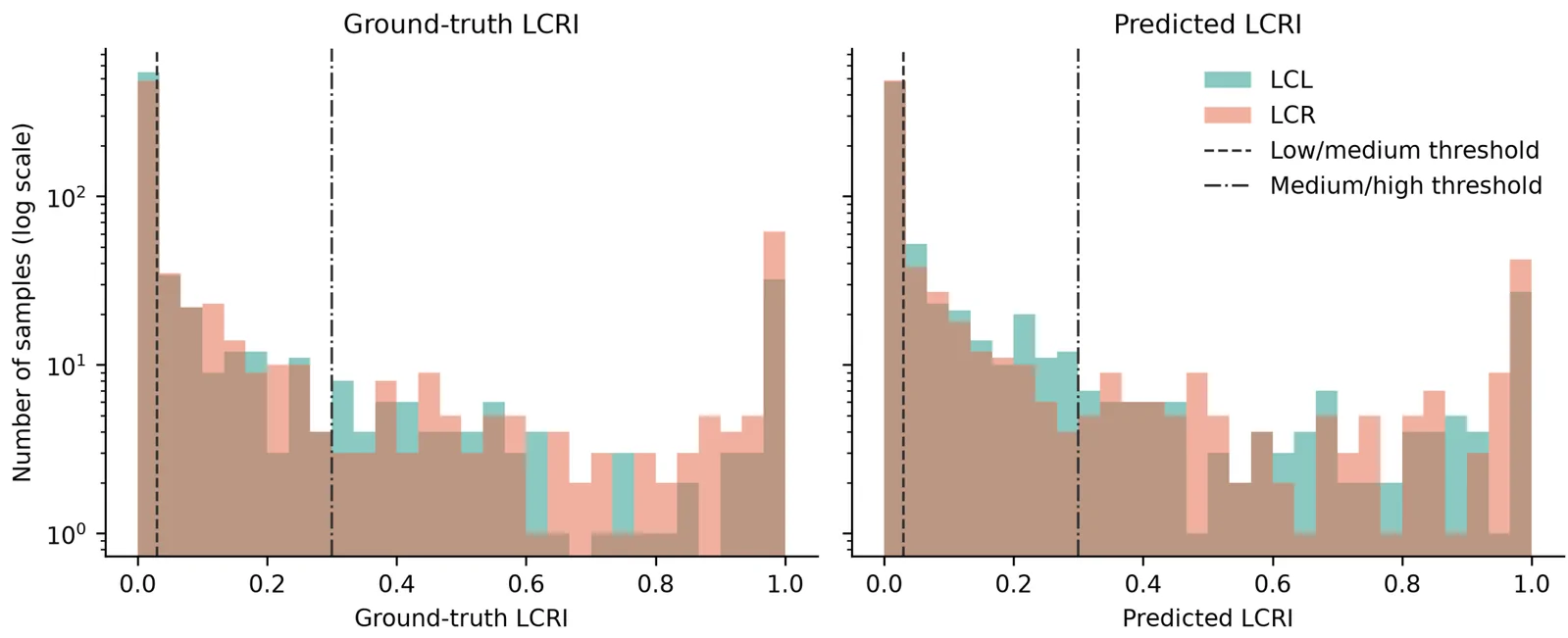
Distribution of ground-truth and forecast-based LCRI values.

With these thresholds, the realized futures contain 1020 low-risk, 246 medium-risk, and 234 high-risk maneuvers. The forecast-based assessment predicts 958, 301, and 241 samples in the three levels, respectively. The confusion matrix in Figure 7 shows that most disagreements occur between adjacent risk levels. Overall, 98.3% of samples fall within one level of the realized-future classification. High-risk maneuvers obtain an F1 score of 0.851, while the medium-risk class obtains an F1 score of 0.702. The LCRI distribution in Figure 8 further shows a continuous and long-tailed risk profile, with high-risk cases reaching LCRI=1. These cases correspond mainly to close-following conflicts during lane changes toward a target lane when a fast-approaching follower is present.

**Figure 9:**
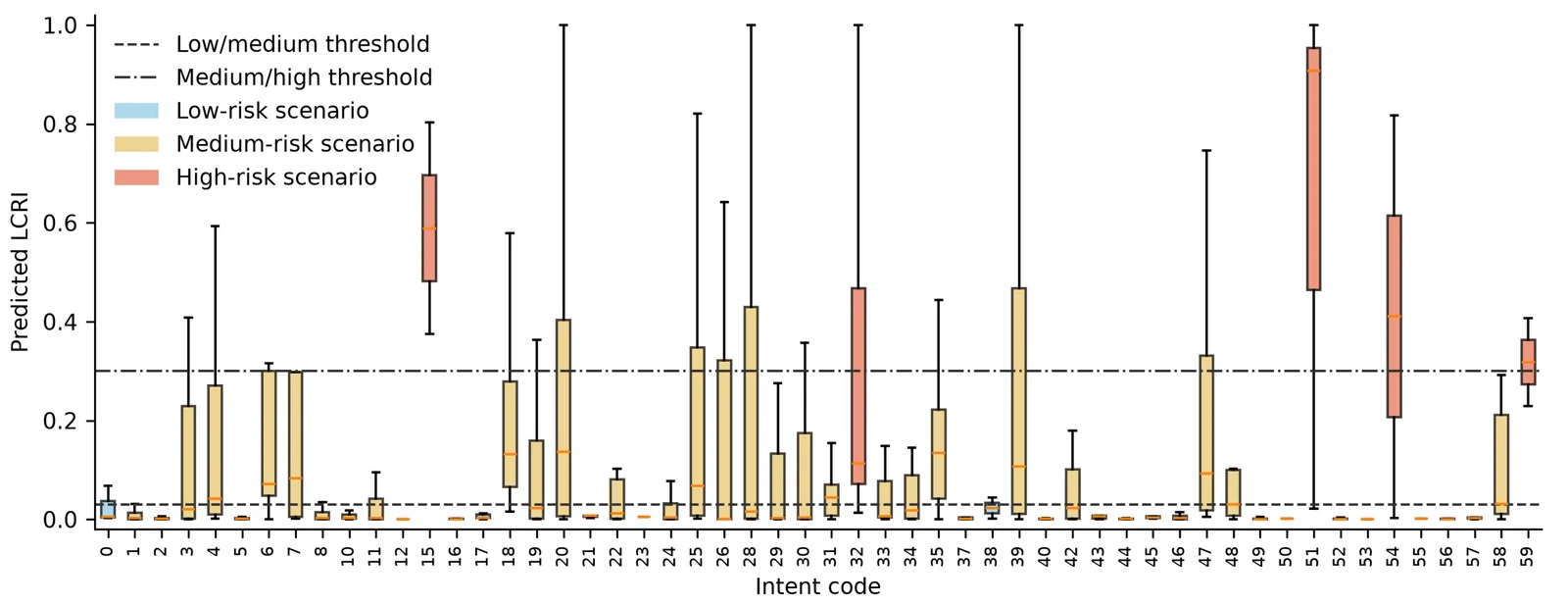
Distribution of the LCRI per intent code.

We further analyze the relationship between discrete intent codes and lane-change risk. Because each forecast is generated under a learned intent code, LCRI values can be grouped by code to examine whether the codebook captures risk-relevant interaction patterns. Of the 60 learned codes, 54 are observed among the 1500 evaluated lane-change samples, with per-code sample counts ranging from 1 to 137 (median 12.5). A total of 18 codes have at least 30 samples and together cover about 80% of the evaluation set. As shown in Figure 9, the per-code mean LCRI ranges from approximately 0.00 to 0.64. The association between codes and risk is both large and statistically significant. The per-code mean LCRI spans a 13-fold range, from 0.022 (code 49, n=45) to 0.285 (code 39, n=85, whose samples exceed the high-risk threshold in 33% of cases), and a Kruskal–Wallis test rejects the hypothesis of a common LCRI distribution across these 18 codes (H=222.7, $p<10^{-37}$, N=1195). This result indicates that the learned codebook captures not only maneuver-level differences but also risk variation within lane-change maneuvers.

## 5. Conclusions

In this paper, we propose IntentDiff, an intent-conditioned diffusion framework for proactive lane-change risk assessment. The framework estimates lane-change risk before the maneuver unfolds by computing LCRI on predicted future trajectories rather than on completed or observed trajectories alone. A vectorized scene context learning module combines a hierarchical VectorNet with a VQ-VAE to map agent–map interactions into discrete intent codes. These codes organize complex traffic situations into interpretable intent prototypes and provide semantic guidance for diffusion-based trajectory generation. Conditioned on the learned intent code, the diffusion model generates kinematically consistent multimodal trajectories that support proactive risk computation.

The experimental findings on the highD dataset can be summarized as follows. First, the framework attains competitive trajectory prediction accuracy, with an ADE of 0.42 m and an FDE of 1.11 m over a 5-s horizon. The sensitivity analysis shows that intent conditioning is responsible for a nearly threefold reduction in displacement error relative to unconditional sampling, while the discrete–continuous conditioning ablation shows that this benefit stems from conditioning itself, whose value lies in interpretability and code-level risk stratification. Second, the learned codebook operates as an effective discrete bottleneck. Sixty codes are active, the codebook perplexity corresponds to roughly 91% capacity utilization, and the codes retain 58% of the maneuver information (I(C;M)=0.63 nats) with an average per-code purity of 0.83. Third, for 1500 naturalistic lane changes, the forecast-based LCRI agrees closely with the realized-future LCRI, indicating that most high-risk lane changes under the surrogate index can be identified before the maneuver is completed, with an end-to-end latency of 45 ms per scenario. These results suggest that the learned codebook captures not only maneuver semantics but also risk-relevant interaction patterns, allowing proactive risk estimates, in the sense of the adopted rear-end surrogate index, to be traced back to interpretable intent prototypes.

Several limitations of this study should be acknowledged. First, since the experiments are conducted on the highD dataset, recorded on structured German highways, the conclusions of this study are limited to structured highway scenarios. Second, the learned codebook characterizes the interaction patterns of highway traffic as represented in highD. Since driving behaviors such as following distance, gap acceptance, and lane-change timing vary across countries and driving cultures, transferring the framework to other regions or unstructured environments, such as urban roads and intersections, would require retraining the codebook on corresponding data. Third, the three-level LCRI thresholds may require recalibration in driving environments with different speed and density profiles. Fourth, the reliability analysis quantifies dataset-level variability through bootstrap resampling and inference-time variability through multiple sampling seeds, but the variability introduced by the training process, including network initialization, mini-batch ordering, codebook initialization, and the stochastic diffusion training, has not been quantified. Therefore, future work will proceed in four directions. First, we will validate the framework on naturalistic trajectory datasets from other countries and road environments. Second, we will extend the framework to more complex urban environments and incorporate joint multi-agent forecasting for surrounding vehicles. Third, we will evaluate the proposed risk index in closed-loop planning to assess its impact on autonomous decision-making. Fourth, the reliability analysis will be extended to the training process through multi-seed training of both stages, together with systematic sweeps of the intent-classification loss weight and the codebook size. 

## Figures and Tables

**Table 1:** Main symbols and tunable parameters of the proposed framework.

Symbol	Description	Value
$T_{obs}$/$T_{pred}$	Observation/prediction horizon	3 s/5 s
*N*	Maximum number of spatial neighbors per scene	8
*Q*/*D*	Codebook size/embedding dimension	60/36
β	Commitment loss weight in $L_{vq}$	0.25
λ	Intent classification loss weight	0→1.0 (warm-up)
*T*	Number of diffusion steps (cosine schedule)	1000
ω	Classifier-free guidance scale	2.0
*p*	Condition drop probability during CFG training	0.1
*K*	Number of generated trajectories per scene	10
Δt	Kinematic integration time step	0.04 s
$N_{mc}$	Number of Monte Carlo trials per interaction	2000

**Table 2:** Information-theoretic characterization of the learned codebook.

Quantity	Value	Reference Bound
Codebook entropy H(C)	3.74	ln60=4.09
Codebook perplexity $e^{H(C)}$	42.1	60
Maneuver entropy H(M)	1.09	ln3=1.10
Conditional entropy H(M∣C)	0.47	–
Mutual information I(C;M)	0.63	H(M)=1.09

Note: *C*: codebook index; *M*: ground-truth maneuver class (LCL, LCR, KL). All entropies in nats.

**Table 3:** Five-second prediction performance of different models.

Model	RMSE (m) over 5 s Prediction Horizon						ADE (m)	FDE@5s (m)
	1 s	2 s	3 s	4 s	5 s	AVG		
S-LSTM	0.22	0.62	1.27	2.15	3.41	1.53	0.62	1.25
CS-LSTM	0.23	0.61	1.24	2.10	3.27	1.49	0.59	1.19
MHA-LSTM (+f)	0.08	0.14	0.31	0.67	1.24	0.49	-	-
iNATran (M)	**0.04**	**0.05**	0.21	0.54	1.11	**0.39**	-	-
DACR-AMTP	0.10	0.17	0.31	0.54	**1.01**	0.42	0.76	1.69
IntentDiff (Ours)	0.09	0.12	**0.18**	**0.52**	1.05	**0.39**	**0.42**	**1.11**

Note: Cells marked with “-” indicate unavailable data. Values shown in bold and underlined highlight the top and second-best performances in each category, respectively.

**Table 4:** Impact of the guidance scale on trajectory prediction performance.

Guidance Scale ω	ADE (m)	FDE@5s (m)
0	1.15	2.85
1	0.68	1.72
2	0.42	1.11
3	0.49	1.28
5	0.75	1.84

**Table 5:** Impact of the number of sampled trajectories on prediction performance.

Samples *K*	ADE (m)	FDE@5s (m)	Inference Time (ms)
1	0.98	2.55	32
5	0.52	1.35	38
10	0.42	1.11	45
15	0.41	1.08	56
20	0.40	1.01	68

**Table 6:** Discrete and continuous conditioning ablation on the test set.

Conditioning	RMSE (m) over 5 s Prediction Horizon						ADE (m)	FDE@5s (m)
	1 s	2 s	3 s	4 s	5 s	AVG		
Ours	0.09	0.12	0.18	0.52	1.05	0.39	0.42	1.11
Continuous variant	0.09	0.11	0.17	0.53	1.07	0.39	0.44	1.05

**Table 7:** Consistency between forecast-based LCRI and realized-future LCRI on 1500 lane-change samples.

Pearson	Spearman	Acc.	W-F1	High Recall
0.886	0.875	0.874	0.878	0.863

**Table 8:** Normal-approximation bootstrap 95% confidence intervals for the LCRI agreement metrics.

Metric	Point Estimate	95% CI
Pearson	0.886	[0.854, 0.918]
Spearman	0.875	[0.853, 0.897]
Accuracy	0.874	[0.858, 0.890]
Weighted F1	0.878	[0.863, 0.894]

**Table 9:** LCRI consistency metrics under *N* = 5 diffusion sampling seeds.

Metric	Mean Across 5 Seeds	Std (Across Seeds)
Pearson	0.886	0.003
Spearman	0.870	0.003
Accuracy	0.870	0.005
Weighted F1	0.874	0.005
High recall	0.856	0.006

## Data Availability

The original contributions presented in this study are included in the article. Further inquiries can be directed to the corresponding author.

## Abbreviations

The following abbreviations are used in this manuscript:

ADE	Average Displacement Error
CFG	Classifier-Free Guidance
CNN	Convolutional Neural Network
CV	Constant Velocity
DDPM	Denoising Diffusion Probabilistic Model
DRAC	Deceleration Rate to Avoid a Crash
FDE	Final Displacement Error
GNN	Graph Neural Network
KL	Keep Lane
LCL	Lane Change Left
LCR	Lane Change Right
LCRI	Lane-Change Risk Index
LSTM	Long Short-Term Memory
MC	Monte Carlo
MLP	Multi-Layer Perceptron
MSE	Mean Squared Error
RCRI	Rear-end Collision Risk Index
RMSE	Root Mean Square Error
SASD	Square of the Absolute Speed Difference
SSM	Surrogate Safety Measure
TTC	Time to Collision
VQ-VAE	Vector Quantized Variational Autoencoder
